# Immunotherapy for small cell lung cancer: the current state and future trajectories

**DOI:** 10.1007/s12672-024-01119-5

**Published:** 2024-08-16

**Authors:** Min Qiang, Hongyang Liu, Lei Yang, Hong Wang, Rui Guo

**Affiliations:** 1 Cancer Center, The First Hospital of Jilin University, Changchun, China; 2 Clinical Laboratory, The First Hospital of Jilin University, Jilin University, Changchun, China

**Keywords:** Immunotherapy, Small cell lung cancer (SCLC), Combined therapy, Bispecific antibodies, ACT, Oncolytic virus, DLL3, TIGIT

## Abstract

Small cell lung cancer (SCLC) constitutes approximately 10% to 15% of all lung cancer diagnoses and represents a pressing global public health challenge due to its high mortality rates. The efficacy of conventional treatments for SCLC is suboptimal, characterized by limited anti-tumoral effects and frequent relapses. In this context, emerging research has pivoted towards immunotherapy combined with chemotherapy, a rapidly advancing field that has shown promise in ameliorating the clinical outcomes of SCLC patients. Through originally developed for non-small cell lung cancer (NSCLC), these therapies have extended new treatment avenues for SCLC. Currently, a nexus of emerging hot-spot treatments has demonstrated significant therapeutic efficacy. Based on the amalgamation of chemotherapy and immunotherapy, and the development of new immunotherapy agents, the treatment of SCLC has seen the hoping future. Progress has been achieved in enhancing the tumor immune microenvironment through the concomitant use of chemotherapy, immunotherapy, and tyrosine kinase inhibitors (TKI), as evinced by emerging clinical trial data. Moreover, a tripartite approach involving immunotherapy, targeted therapy, and chemotherapy appears auspicious for future clinical applications. Overcoming resistance to post-immunotherapy regimens remains an urgent area of exploration. Finally, bispecific antibodies, adoptive cell transfer (ACT), oncolytic virus, monotherapy, including Delta-like ligand 3 (DLL3) and T cell immunoreceptor with Ig and ITIM domains (TIGIT), as well as precision medicine, may present a prospective route towards achieving curative outcomes in SCLC. This review aims to synthesize extant literature and highlight future directions in SCLC treatment, acknowledging the persistent challenges in the field. Furthermore, the continual development of novel therapeutic agents and technologies renders the future of SCLC treatment increasingly optimistic.

## Introduction

Lung cancer has emerged as a predominant global health challenge, representing the second most frequently diagnosed malignancy and the principal cause of cancer-related mortality in 2020. Specifically, it accounted for approximately 11.4% of all cancer diagnoses and 18% of all cancer-related deaths [1]. Lung cancer can be classified into two distinct subtypes: small cell lung cancer (SCLC) and non-small cell lung cancer (NSCLC) [2].

SCLC constitutes approximately 10–15% of all lung cancer cases and is associated with high mortality rates [3]. SCLC derives its nomenclature from the microscopic observation of its small, oval-shaped cellular morphology. This malignancy is distinguished by its rapid proliferative kinetics and its propensity for swift metastasis to distant sites. Notably, therapeutic options remain limited and largely ineffective upon disease recurrence [4, 5].

According to the differential expression levels of specific genes, SCLC subtypes are classified as follows: those with elevated achaete-scute homolog 1 (ASCL1) expression are designated SCLC-A; those with increased neurogenic differentiation factor 1 (NEUROD1) expression fall under SCLC-N; elevated POU class 2 homeobox 3 (POU2F3) expression delineates SCLC-P; and those with heightened yes-associated protein 1 (YAP1) expression are classified as SCLC-Y. These taxonomical distinctions underscore the inherent heterogeneity of SCLC and pave the way for the development and application of subtype-specific targeted therapies [6]. Owing to the complex landscape of tumor heterogeneity in SCLC, there is a pressing imperative to identify and validate additional biomarkers. This endeavor is not merely an academic exercise, but serves to stratify the patient population most amenable to immunotherapeutic interventions. It also facilitates more rigorous evaluation of immunotherapeutic efficacy, thereby enhancing the precision of therapeutic regimens in SCLC [7–9].

Clinically, SCLC is categorized into two stages: limited-stage and extensive-stage. The malignancy is characterized by its aggressive nature and poor prognosis. The majority of patients are diagnosed with extensive-stage disease at the time of initial presentation [10]. The term "extensive-stage" denotes that the malignancy has metastasized beyond the lungs to other regions of the body.

Standard treatment modalities for SCLC commonly incorporate a regimen of chemotherapy and radiation therapy, with surgical intervention considered in select cases. Over the past decade, chemotherapy has persisted as the cornerstone for both first- and second-line therapies across the spectrum of SCLC [11]. In this context, limited-stage small cell lung cancer (LS-SCLC) predominantly relies on a treatment strategy of radiotherapy coupled with chemotherapy. For extensive-stage small cell lung cancer (ES-SCLC), chemotherapy serves as the principal therapeutic approach. Specifically, a combination of etoposide with either cisplatin or carboplatin has remained the standard chemotherapy regimen for ES-SCLC for over three decades [12]. While initial treatment often results in some degree of remission for patients with SCLC, the propensity for disease relapse and progression is high, and therapeutic options following relapse are limited. Consequently, the 5-year survival rate remains suboptimal, which OS is only about 2%. Despite minimal advancements in overall survival (OS) over the years, contemporary literature has increasingly focused on the promise of immunotherapy. Immune checkpoint blockages (ICBs), particularly programmed cell death protein 1/programmed death-ligand 1 (PD-1/PD-L1) inhibitors such as atezolizumab and durvalumab, are currently the subject of rigorous investigation (Table 1) [13]. The advent of immunotherapy has marked a seminal shift in the treatment landscape for SCLC, achieving for the first time an OS exceeding 1 year and ushering SCLC into the immunotherapeutic era [14]. Nonetheless, PD-1 inhibitors have not achieved a significant breakthrough in the treatment of SCLC. Insights from the IMpower133 and CASPIAN trials indicate that PD-L1 monoclonal antibodies have demonstrated incremental advancements [15]. To date, the National Medical Products Administration (NMPA) has granted approval for the use of atezolizumab, durvalumab, serplulimab, and adebrelimab in the first-line treatment of ES-SCLC [15–18]. Nonetheless, the clinical trials and resultant data for durvalumab and serplulimab have not yet received approval from the U.S. Food and Drug Administration (FDA). Recently, immunotherapy has gained approval as a first-line treatment for metastatic SCLC in combination with chemotherapy. It has also been sanctioned as a third-line treatment for metastatic SCLC following the unsuccessful outcomes of two prior chemotherapy regimens [16].

**Table 1:** Various ICB drugs and their targets

Target/Bioactivity	ICB drugs
Anti-CTLA-4	Ipilimumab
	Tremelimumab
Anti-PD-1	Nivolumab
	Tislelizumab
	Toripalimab
	Sintilimab
	Camrelizumab
	Serplulimab
	Pembrolizumab
Anti-PD-L1	Atezolizumab
	Durvalumab
	Adebrelimab

In recent years, there have been tremendous changes in the treatment of SCLC. Multiple treatment modalities have been applied to enhance patient survival outcomes. This paper provides a comprehensive overview of the utilization of immunotherapy in concert with chemotherapy for the treatment of SCLC. The basic information on the clinical trials discussed in this article can be found in Table 2. This review aims to investigate the existing circumstances in the treatment of SCLC and provide directions in future treatments. Therefore, the study serves as a seminal contribution to the existing literature on the trajectories and future prospects of immunotherapy combined with chemotherapy in SCLC within the realm of clinical research. This study has provided fresh insights and perspectives on the treatment of SCLC. Our study facilitates a deeper understanding of developing new therapeutic strategies aimed at significantly improving the patient-survival rate.

**Table 2:** Ongoing clinical trials investigating immunotherapy in SCLC

Trail ID	Drug	Phase	Cancer stage	Comments
NCT04471727	HPN328, atezolizumab	I/II	SCLC	
NCT01928394	Nivolumab, ipilimumab	I/II	SCLC	
NCT05505825	AK104 IV infusion, chiauranib	I/II	ES-SCLC	
NCT02763579	Atezolizumab, carboplatin, etoposide	I/III	ES-SCLC	Improved OS and a tolerable safety profile
NCT02402920	Pembrolizumab, thoracic radiotherapy (TRT)	I	LS-SCLC	
NCT05116007	AK112, etoposide, carboplatin	I	ES-SCLC	
NCT05680922	LB2102	I	ES-SCLC	
NCT05507593	DLL3-CAR-NK cells	I	ES-SCLC	
NCT05652686	PT217	I	SCLC	
NCT04429087	BI 764532	I	SCLC	The maximum tolerated dose of BI 764532 monotherapy
NCT03319940	Tarlatamab, pembrolizumab, CRS Mitigation Strategies	I	SCLC	Manageable safety with encouraging response durability
NCT05780307	IMM2520	I	NSCLC, SCLC	
NCT04952597	Ociperlimab, tislelizumab, concurrent chemoradiotherapy	II	LS-SCLC	
NCT05384015	Pembrolizumab, lenvatinib, carboplatin, etoposide	II	ES-SCLC	
NCT04933175	Fluzoparib, anlotinib hydrochloride, platinum	II	ES-SCLC	Increase the clinical benefit
NCT04063163	Serplulimab, placebo, carboplatin, toposide	III	ES-SCLC	
NCT03066778	Pembrolizumab, placebo, etoposide, platinum	III	ES-SCLC	Improved PFS
NCT02538666	Nivolumab, ipilimumab	III	ES-SCLC	Not prolong OS
NCT04005716	Tislelizumab, placebo, platinum, etoposide	III	ES-SCLC	
NCT00546130	Irinotecan hydrochloride, cisplatin, krestin	III	ES-SCLC	
NCT03711305	Adebrelimab, placebo, carboplatin, etoposide	III	ES-SCLC	Significantly improved OS with an acceptable safety profile
NCT03043872	Durvalumab,tremelimumab, platinum-etoposide	III	ES-SCLC	
NCT04256421	Tiragolumab, atezolizumab, carboplatin, etoposide, placebo	III	SCLC	

## Related countries and institutions of this field

The United States and China emerge as the leading nations in the volume of scholarly publications focused on SCLC. In the context of epidemiological burden, lung cancer is the second most frequently diagnosed malignancy in the United States. Within this cadre, SCLC constitutes approximately 13% of all diagnosed lung carcinomas [19]. In the realm of SCLC research, the contributions from the United States are salient, bolstered by cutting-edge technological advancements and a cadre of distinguished experts in the field. Other developed nations also make significant scholarly contributions, reflecting a robust international effort in this area. China, with its expansive population, contributes substantially to the pool of available clinical trials and patient cohorts. It is noteworthy that SCLC patients from Western nations exhibit distinct molecular and clinical characteristics. Conversely, the phenotypic and molecular profiles of Chinese SCLC patients warrant more comprehensive investigation for tailored therapeutic approaches [20]. Memorial Sloan Kettering Cancer Center, the world's preeminent private cancer research institution, and Bristol-Myers Squibb stand as two paradigmatic entities within the United States, each wielding considerable influence in the oncologic sphere. These institutions have been instrumental in advancing our understanding of cancer etiology, diagnostics, and therapeutics. Concurrently, Shanghai Jiao Tong University and Harbin Medical University in China serve as leading academic centers, propelling the medical industry forward through rigorous research and innovation.

## ICBs combine with somatostain receptors (SSTR)

Immune Checkpoint Blockades (ICBs) function via a series of well-characterized molecular mechanisms to modulate immune responses. Specifically, they target inhibitory checkpoint molecules such as cytotoxic T-lymphocyte antigen 4 (CTLA-4), PD-1, and PD-L1, which are integral to the preservation of self-tolerance—a biologically requisite safeguard that mitigates autoimmune destruction of host tissue [21]. Given the burgeoning applicability of immunotherapeutic approaches across a diverse array of malignancies, these inhibitory checkpoints have emerged as compelling therapeutic targets [22]. These therapeutic interventions act by disrupting the molecular interactions between immune checkpoint proteins and their respective ligands. This disruption abrogates the inhibitory signaling cascade, thereby facilitating the activation and effector functions of T lymphocytes in targeting neoplastic cells. To illustrate the expression of the inhibitory checkpoint receptor PD-1 on CD8+ T lymphocytes, or its cognate ligand PD-L1 on tumor or immune cells, attenuates T cell effector functions, blocking this PD-1/PD-L1 interaction and consequently it mitigates T cell exhaustion and augments the host's immunological efficacy against malignant cells [23–25]. MicroRNAs (miRNAs) are small non-coding RNAs that could regulate gene expression. It plays a vital role in modulating both PD-1/PD-L1 and CTLA-4 immune checkpoints. It is essential to understand the interaction between miRNAs and immune checkpoints, which could provide valuable insights into developing more effective and personalized immunotherapy strategies for SCLC treatment [26].

In clinical investigations assessing immune checkpoint inhibitors, diverse pharmacological agents have been designed to modulate distinct molecular pathways within the immune milieu. To illustrate, nivolumab and pembrolizumab have received regulatory approval as antagonistic monoclonal antibodies targeting the PD-1 receptor. In parallel, atezolizumab and durvalumab have garnered authorization as inhibitors of PD-L1. These pharmacologic agents operate by attenuating the interactions between PD-1 and PD-L1, thereby ameliorating immune suppression and potentiation of anti-tumor immune responses, consequently impairing the neoplastic cells' ability to elude immunological surveillance [27, 28]. Ipilimumab and tremelimumab, classified as antagonistic monoclonal antibodies targeting the CTLA-4 receptor, have demonstrated significant antitumor efficacy, thereby prolonging overall survival in patients afflicted with SCLC [29].

In an endeavor to ameliorate patient survival rates, ICBs are increasingly being employed in therapeutic regimens concomitant with established modalities such as chemotherapy, radiotherapy, and targeted therapies. Specifically, within the context of ES-SCLC, immunotherapy has been inducted into the first-line treatment algorithm, marking a paradigmatic shift in contemporary therapeutic strategies [30]. Pembrolizumab and Nivolumab, the most frequently cited agents, are both PD-1 monoclonal antibodies. These PD-1-targeted agents have found expansive application in the therapeutic landscape of SCLC, with pembrolizumab demonstrating noteworthy antitumor efficacy, particularly in patients harboring pretreated, PD-L1 expressing SCLC. ICBs have seen a diversification in their application, not only as first-line agents but also in maintenance therapies and subsequent lines of treatment. Of note, the integration of either atezolizumab or durvalumab with platinum-based chemotherapy stands as the sole regimen that has demonstrated a statistically significant augmentation in overall survival, concomitantly improving the quality of life for patients [31]. However, it must be acknowledged that therapeutic efficacy remains circumscribed to a minority of patients. Consequently, a plethora of research avenues remain to be explored, encompassing the determination of optimal treatment regimens and the identification of additional biomarkers for targeted interventions [14].

A seminal article, cited extensively within the medical literature, posits an intriguing hypothesis—namely, that NSCLC and SCLC may indeed originate from the same progenitor cells [32]. Contrary to the conventional paradigm, which stipulates distinct lineages for these two subtypes, multiple case reports have substantiated their coexistence. The implications of this are profound, especially as it opens the avenue for the application of therapeutic modalities, heretofore specific to NSCLC, to also be considered for SCLC treatment [32]. It is noteworthy that the majority of ICBs predominantly target the PD-1 and PD-L1 pathways, with several PD-L1 Immunohistochemistry (IHC) assays being employed to guide drug prescriptions in both first-line and second-line treatment settings [33]. In contrast to NSCLC, a significant proportion of patients experiencing relapse within the initial two years after diagnosis encounter limited alternatives in the context of second-line therapy for SCLC [34, 35]. Historically, topotecan served as the sole second-line pharmacotherapeutic modality for patients with ES-SCLC, boasting an objective response rate (ORR) of 22–24%. Nonetheless, the therapeutic landscape experienced a paradigm shift in June 2020, following the Food and Drug Administration's approval of Lurbinectedin as a second-line regimen for metastatic SCLC. Concurrently, emerging data suggest that apatinib may hold promise as an alternative treatment option, particularly in refractory cases failing second-line or multiline chemotherapy, with reported ORRs ranging from 13.6% to 17.5% [35].

Tumor Mutational Burden and Efficacy of Nivolumab Monotherapy and in Combination with Ipilimumab in Small-Cell Lung Cancer (NCT01928394).

Whole-exome sequencing was utilized to scrutinize the influence of tumor mutational burden (TMB) on the therapeutic responsiveness to nivolumab monotherapy as well as its combinatorial regimen with ipilimumab. In both therapeutic cohorts—namely, the nivolumab monotherapy and the nivolumab plus ipilimumab arms—patients manifesting high TMB levels (21.3% and 46.2% objective response rates, respectively) exhibited statistically superior treatment outcomes relative to those with low (4.8% and 22.2% objective response rates, respectively) or intermediate TMB levels (6.8% and 16.0% objective response rates, respectively). It is noteworthy that high TMB levels were associated with better response rates for both nivolumab monotherapy and the combination with ipilimumab. In particular, among patients categorized in the upper tertile of TMB, the combination of nivolumab and ipilimumab conferred a discernible advantage in terms of clinical benefit over nivolumab monotherapy [36]. Commonly, nivolumab and ipilimumab were combined, showing greater efficacy than either agent alone.

Recent investigations have accentuated the suboptimal therapeutic efficacy of immune monotherapy in the context of SCLC, predominantly endorsing its deployment as maintenance therapy subsequent to initial treatment with immunotherapy concomitant to chemotherapy, with or without radiotherapy. In light of these findings, the role of ICBs in the SCLC therapeutic landscape is indubitably poised to be a cornerstone in future scientific inquiry and clinical strategies. This purview encompasses, but is not circumscribed to, the assimilation of ICBs into multimodal treatment paradigms.

## ICBs combined with chemotherapy

**Fig. 1:**
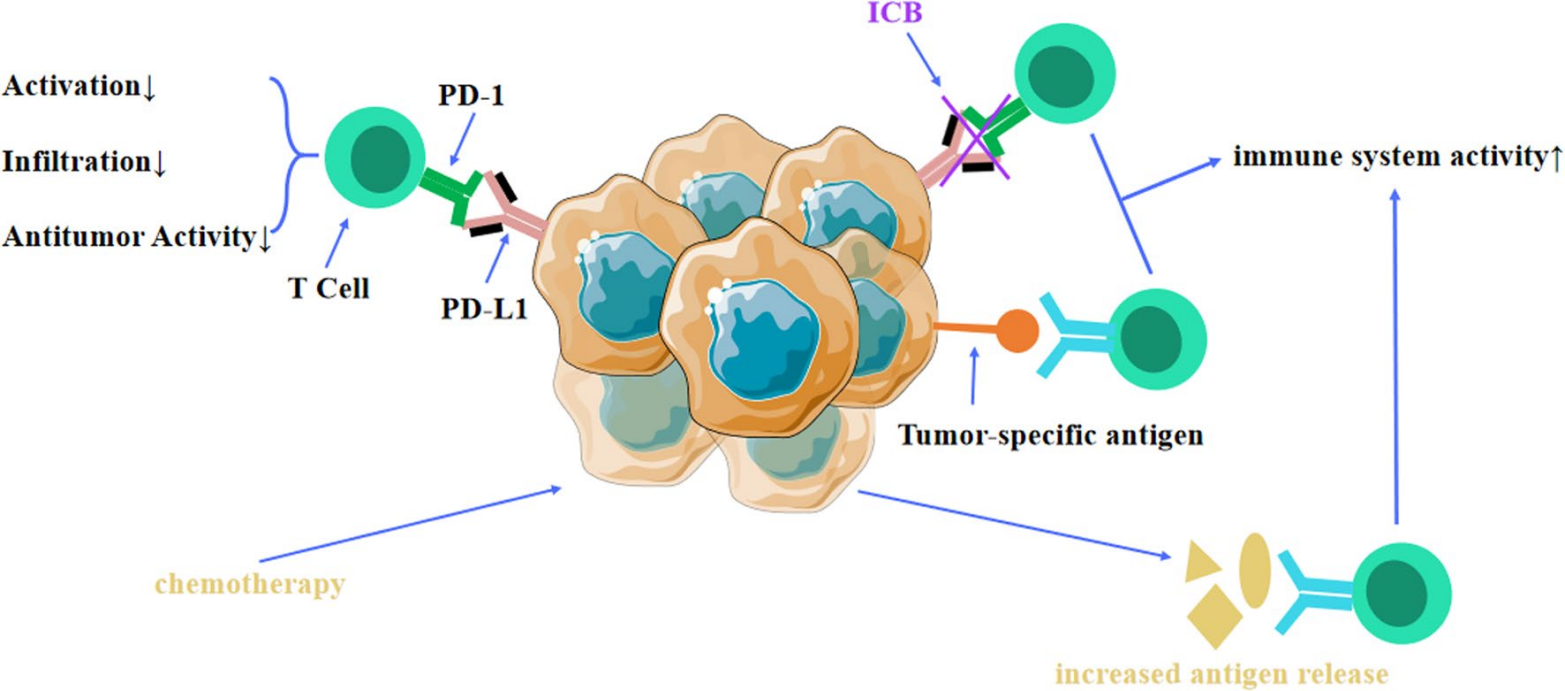
Tumor cells eliminate tumor cells by inhibiting T cells through the PD-L1/PD-1 axis. ICB blocks the PD-L1/PD-1 axis, allowing T cells to recognize tumor cell receptors. Chemotherapy kills tumor cells and releases intracellular antigens, further improving the effectiveness of immunotherapy by helping T cells recognize antigens

Conventional chemotherapy, historically employed as a first-line therapeutic intervention for SCLC, has demonstrated suboptimal antitumor efficacy, often failing to achieve durable remissions [37, 38]. This limitation may be attributable to the ability of SCLC cells to engender a specialized tumor microenvironment (TME); notably, the adhesion of these malignant cells to the extracellular matrix (ECM) has been implicated in amplifying tumorigenicity and conferring resistance to chemotherapeutic agents [39]. Many herbal products that attract great attention in cancer treatment due to considerable potential in anti-tumor effects and reducing the side effects of chemotherapy. Nanoparticle (NP) functions as a carrier is applied in the chemotherapy, which can increase the intracellular concentrations of drugs and enhance synergistic effects in SCLC therapy [40–46]. Recent advancements in oncologic therapeutics have revealed the promising potential of immunotherapy in the management of ES-SCLC [47]. Specifically, immunotherapy has been demonstrated to potentiate the host's immunological recognition of neoplastic cells and to fortify 'immune memory,' thereby affording a subset of patients durable remissions and diminished relapse rates post-medication [48–50]. It is currently believed that this is related to chemotherapy-induced immunogenic cell death in tumor cells (Fig. 1). However, the clinical efficacy of ICBs in synergy with chemotherapy has yielded only incremental improvements in OS in the ES-SCLC cohort, highlighting their circumscribed therapeutic benefit [51].

The incipient foray into the domain of immunotherapeutic interventions for recurrent SCLC can be dated to 2007, as substantiated by clinical trial NCT00546130. This pivotal trial adopted a regimen incorporating Irinotecan hydrochloride, cisplatin, and krestin for the treatment of patients with ES-SCLC. Krestin—clinically known as Polysaccharide K or PSK—is an adjuvant extracted from the coriolus versicolor mushroom. Its application as an adjuvant immunotherapeutic agent is not novel; indeed, it enjoys widespread use in Japan for the treatment of various malignancies, inclusive of lung cancer [52]. While the initial trial in 2007 served as a rudimentary amalgamation of chemotherapy and immunotherapy, the pace of progress in this domain has a sustainable grow in the subsequent years.

Of particular note, there were several researches examined ICBs in conjunction with chemotherapy, revealing that both atezolizumab and durvalumab, when administered alongside chemotherapy, significantly improved overall survival without introducing new adverse events [53, 54]. However, it merits mention that the synergy of ipilimumab with chemotherapy did not yield enhanced efficacy, possibly due to the unique pharmacological profiles of the agents involved. While compelling, these findings suggest that further clinical trials are requisite for more conclusive evidence. The two most frequently cited agents—pembrolizumab and nivolumab—are PD-1 monoclonal antibodies, demonstrating superior outcomes in patients with elevated PD-L1 expression levels. The pinnacle of citations was achieved by a 2018 article on first-line atezolizumab plus chemotherapy in ES-SCLC; by March 2019, FDA conferred approval upon the PD-L1 inhibitor atezolizumab as a component of a first-line therapeutic regimen inclusive of etoposide and carboplatin, underscoring its unparalleled potential in the field, with additional trials presently underway. Subsequently, in 2020, the FDA similarly endorsed durvalumab in combination with either etoposide and cisplatin or carboplatin for initial treatment of ES-SCLC. Comparative analysis revealed that the median OS for patients administered these combined modalities witnessed an increment of 2–3 months relative to chemotherapy monotherapy in the same patient population.

The integration of ICBs with chemotherapy constitutes a paradigm shift in the management of ES-SCLC. Particularly, atezolizumab is frequently utilized in concert with chemotherapy for those patients who demonstrate a partial response to initial chemotherapy. While agents targeting the PD-1 axis have lagged behind their PD-L1 counterparts in clinical application for SCLC, they are steadily gaining traction. Pembrolizumab, an anti-PD-1 agent, was initially deployed in 2015 as monotherapy for advanced solid tumors. Its use has since been expanded to include combination regimens with chemotherapy or targeted therapies for recurrent or metastatic SCLC. Notably, serplulimab, another anti-PD-1 agent, received approval from the NMPA in 2022 for its synergistic role with first-line chemotherapy in ES-SCLC, following the auspicious outcomes of the phase III ASTRUM-005 clinical trial (NCT04063163) [55]. Adebrelimab, an anti-PD-L1 immune checkpoint inhibitor, acts by selectively binding to PD-L1 molecules, thus inhibiting the PD-1/PD-L1 interaction that otherwise facilitates immune tolerance within the TME. By interrupting this inhibitory pathway, the drug reinvigorates the anti-tumoral immune responses, offering a therapeutic advantage in the management of ES-SCLC. Clinical validation of adebrelimab's efficacy comes from the CAPSTONE-1 study (NCT03711305), a randomized, double-blind, phase III clinical trial, which demonstrated favorable outcomes. As a testament to its emerging role in oncologic therapeutics, adebrelimab received approval from NMPA in 2023 [56]. Camrelizumab, primarily investigated in conjunction with apatinib and traditional chemotherapy, is the subject of seven clinical trials aimed at elucidating its therapeutic potential in the management of SCLC. As for sintilimab, it is featured in seven clinical investigations, yet the outcomes remain undisclosed at this juncture. Similarly, toripalimab is under evaluation in six clinical trials, but the results are pending. Tislelizumab is in the early stages of clinical investigation, being a component of five trials, all confined to phase I and phase II studies; these trials are also awaiting the release of outcomes.

Updated Overall Survival and PD-L1 Subgroup Analysis of Patients With Extensive-Stage Small-Cell Lung Cancer Treated With Atezolizumab, Carboplatin, and Etoposide (IMpower133: NCT02763579).

The IMpower133 trial stands as a seminal Phase III study that constitutes the inaugural utilization of first-line immunotherapy to achieve encouraging results in ES-SCLC, thereby serving as a landmark investigation in this clinical context. This study heralded the first evidence of enhanced OS in patients with ES-SCLC subjected to combinatorial immunotherapeutic regimens predicated on immune checkpoint inhibitors, marking a milestone by elevating the median OS to exceed 1 year for the first time [57]. The results disclosed a median OS of 12.3 months for the atezolizumab arm, compared to 10.3 months in the standard chemotherapy cohort, a discrepancy bearing statistical significance (HR = 0.82, P = 0.008) [12]. Expanding upon this, the Phase IV IMbrella study—encompassing survivors from the initial IMpower133 trial—revealed unprecedented 5-year survival metrics. Specifically, ES-SCLC patients administered a combined regimen of atezolizumab and chemotherapy exhibited a 5-year OS rate of 12%, a six-fold augmentation compared to the 5-year OS rate of 2% observed in patients relegated to chemotherapy monotherapy, thereby underlining the substantial survival benefits conferred by the addition of atezolizumab.

Durvalumab, with or without tremelimumab, plus platinum-etoposide versus platinum-etoposide alone in first-line treatment of extensive-stage small-cell lung cancer (CASPIAN): updated results from a randomised, controlled, open-label, phase 3 trial (NCT03043872).

In a comparative evaluation, the study investigated the therapeutic efficacies of durvalumab in conjunction with chemotherapy versus standard chemotherapy, specifically carboplatin and etoposide, in a cohort of patients diagnosed with ES-SCLC. The analysis delineated that the median OS in the durvalumab-combined chemotherapy arm was 12.9 months, juxtaposed against a median OS of 10.5 months in the standard chemotherapy cohort. This variance manifested statistical significance, corroborated by a Hazard Ratio (HR) of 0.75 and a p-value of 0.0032, thereby indicating the incremental survival benefits associated with the durvalumab regimen [47].

Effect of first-line serplulimab vs placebo added to chemotherapy on survival in patients with extensive-stage small cell lung cancer: The ASTRUM-005 Randomized Clinical Trial (NCT04063163).

The ASTRUM-005 randomized clinical trial embarked upon a rigorous assessment of both the therapeutic efficacy and safety profile associated with the integration of the PD-1 inhibitor, serplulimab, into the standard chemotherapy regimen for first-line treatment in patients afflicted with ES-SCLC. The findings unambiguously demonstrated a protracted median OS in the serplulimab-augmented cohort, registering at 15.4 months, vis-a-vis a median OS of 10.9 months in the placebo-controlled arm. This differential in survival outcomes reached unequivocal statistical significance, as substantiated by a HR of 0.63 and a p-value less than 0.001 [55].

Adebrelimab or placebo plus carboplatin and etoposide as first-line treatment for extensive-stage small-cell lung cancer (CAPSTONE-1): a multicentre, randomised, double-blind, placebo-controlled, phase 3 trial (NCT03711305).

The CAPSTONE-1 clinical trial rigorously evaluated the therapeutic efficacy and safety profile of adebrelimab, an anti-PD-L1 monoclonal antibody, when conjoined with standard chemotherapy regimens for initial treatment in patients diagnosed with ES-SCLC. The median OS was conspicuously superior in the adebrelimab-cohort, registering 15.3 months, as opposed to 12.8 months in the placebo-controlled arm, thus achieving statistical significance with a HR of 0.72 and a p-value of 0.0017 [56].

Subsequently, the NMPA has authorized four immune checkpoint inhibitors—atezolizumab, durvalumab, serplulimab, and adebrelimab—for frontline treatment in ES-SCLC [15, 55–57]. Collectively, the survival advantage conferred by the triad of PD-L1 inhibitors oscillates between 2 to 2.5 months, with highly analogous HR metrics indicating approximately a 25% decrement in mortality risk. Notably, serplulimab, the sole PD-1 inhibitor approved for ES-SCLC, manifested an OS of 15.8 months—translating into a marked survival elongation of 4.7 months vis-a-vis standard chemotherapy and a consequential 38% reduction in mortality risk.

The protocols employed in IMpower133 [57], CASPIAN2 [15], ASTRUM-0053 [55] and CAPSTONE-1 [56] share overarching congruencies. It's worth emphasizing that while ASTRUM-005 was an international multicenter phase III study employing a randomized, double-blind, placebo-controlled design akin to the IMpower133 trial, CAPSTONE-1 was conducted exclusively within China, involving only Chinese patients. This latter dimension warrants further investigation to discern whether the observed outcomes are inextricably linked to demographic peculiarities endemic to the Chinese population.

In the realm of ICBs studies in ES-SCLC, it is imperative to delineate those clinical trials that failed to meet their primary endpoints, as this serves to enrich our collective understanding of therapeutic limitations. Two such studies warrant discussion: KEYNOTE-604 and CheckMate 451. In the KEYNOTE-604 investigation, the co-administration of pembrolizumab with etoposide and platinum (EP) yielded an extended OS; however, the improvement failed to cross the threshold of statistical significance, as evidenced by a HR of 0.80 and a p-value of 0.0164. The 24-month OS estimates further corroborated this observation: 22.5% in the pembrolizumab plus EP arm versus 11.2% in the placebo plus EP arm (NCT03066778) [58]. Similarly, in the CheckMate 451 trial, the combination of nivolumab and ipilimumab did not confer a statistically significant survival benefit, as indicated by an HR of 0.92 and a p-value of 0.37. Intriguingly, nivolumab alone yielded a somewhat favorable HR of 0.84 vis-a-vis placebo, though the median OS registered at 10.4 months (NCT02538666) [59].

Furthermore, the confluence of chemotherapy and immunotherapy—commonly termed chemo-immunotherapy—has engendered incremental survival benefits in SCLC cohorts, albeit with persistently suboptimal outcomes for the majority of patients. Thus, further elucidation of immune inhibitory and stimulatory pathways heralds myriad opportunities for future advancements in the field.

## ICBs combined with targeted therapy

In the contemporary landscape of oncological therapeutics, the amalgamation of immunotherapy and targeted agents has emerged as a transformative paradigm, eliciting considerable enthusiasm for its potential to revolutionize the standard of care in various malignancies. While single-agent strategies for ES-SCLC remain under rigorous investigation, they are constrained by modest efficacy and a non-negligible profile of adverse events [60]. There exist four principal molecular targets that have garnered attention in this context: tyrosine kinase inhibitors (TKIs), vascular endothelial growth factor receptors (VEGFR), poly ADP-ribose polymerase (PARP), and somatostatin receptors. Each of these targets is represented by multiple pharmacologic agents (Table 3), rendering the therapeutic armamentarium increasingly versatile.

**Table 3:** Targeted antibodies for lung cancer therapy

Targeted antibodies	Target/Bioactivity	Related molecule
Bispecific antibodies	Anti-PD-1, Anti-CTLA-4	Cadizumab
	Anti-PD-1, Anti-VEGF	AK112
	Anti-PD-L1, Anti-CD47	IMM2520
	Anti-DLL3, Anti-CD47	PT217
	Anti-DLL3, Anti-CD3	BI 764532
	Anti-PD-1, Anti-TGF-β	JS201
	Anti-PD-L1, Anti-TGF-β	M7824
	Anti-PD-L1, Anti-VEGF	PM8002
Vaccine	Anti-p53	INGN-225
Antibody–drug conjugates (ADCs)	Anti-CD30	Brentuximab vedotin
Monotherapy	Anti-DLL3	Rova-T
	Anti-DLL3	AMG119
	Anti-DLL3	Tarlatamab
	Anti-DLL3	HPN328
	Anti-TIGIT	Tiragolumab
	Anti-TIGIT	OMP-313M3
	Anti-TIGIT	Ociperlimab

### ICBs combine with somatostain receptor (SSTR)

The elucidation of somatostatin receptors (SSTR 1–5) and their ligands, notably [177Lu] Lu-DOTA-TATE and [68 Ga] Ga-DOTA-TATE, marks a seminal advancement in the realm of targeted cancer therapeutics [61]. The clinical translation of these somatostatin analogues has evinced considerable promise in the oncologic arena [62]. The conceptual framework of exploiting the DNA damage response (DDR) pathways for anti-tumor immunity adds another layer of complexity and opportunity. A particular point of interest lies in the synergistic potential of DDR inhibitors with PD-L1 inhibitors to augment cytotoxic T cell infiltration within the TME of SCLC. Additionally, DDR inhibitors have been shown to act as instigators of the interferon gene (STING)/TANK-binding kinase 1 (TBK1)/interferon regulatory transcription factor 3 (IRF3) innate immune axis, culminating in the elevated expression of chemokines such as CXCL10 and CCL5. These chemokines, in turn, play an instrumental role in modulating the activation and effector function of cytotoxic T lymphocytes [63].

### CKB combine with TKI and chemotherapy

A phase II clinical trial which started in November 2022, is ongoing (NCT05384015). The confluence of pembrolizumab, lenvatinib, and conventional chemotherapeutic agents in the first-line treatment of ES-SCLC offers a new frontier in oncologic research. The study employs a dual-phase approach, with Part 1 concentrating on assessing the safety profile of this therapeutic triad. This aligns with conventional early-phase clinical trial designs that prioritize the evaluation of adverse events and maximum tolerated dose. In Part 2, the primary focus shifts to progression-free survival, evaluated through the rigorous RECIST 1.1 criteria. This phase aims to provide more comprehensive data on the efficacy of the treatment regimen, thus advancing our understanding of its potential clinical utility (NCT04933175). The recruitment of a cohort of 50 patients to assess the objective response rate as the primary endpoint aligns with the statistical rigors often required in phase II clinical trials [64]. The subsequent inquiry into the efficaciousness and safety profile of anlotinib in conjunction with platinum-based chemotherapy yields promising outcomes, notably a median progression-free survival (PFS) of 10.3 months and a median OS of 17.1 months [65]. Anlotinib has garnered approval from the NMPA, distinguishing it from other pharmacologic agents like donafenib, famitinib, lenvatinib, and surufatinib that remain in the experimental phase. A pioneering multicenter, placebo-controlled, randomized phase III clinical study evaluates the combinatorial efficacy of benmelstobart and anlotinib alongside a chemotherapy regimen (NCT04005716). This therapeutic amalgamation aims to reshape the TME and augment immune cell infiltration. The study evidences a profound impact on key clinical indicators: median PFS (6.9 months), median OS (19.3 months), objective response rate (81.3%), and duration of response (5.8 months). Remarkably, the safety profile corroborates the regimen's tolerability, setting a new precedent with the longest documented overall survival and progression-free survival in the context of ES-SCLC [66].

### ICBs combine with TKIs

Tyrosine kinase inhibitors (TKIs), such as anlotinib, apatinib, chiauranib, famitinib, lenvatinib, and surufatinib, serve as pharmacologic disruptors of protein kinase signal transduction pathways via various mechanisms of inhibition [35, 67–69]. Among them, anlotinib, a targeted therapeutic agent, emerged as the preeminent focus, often deployed in concert with immunotherapeutic strategies, representing a notable advancement in SCLC treatment paradigms. Apatinib, a small-molecule TKI, selectively abrogates vascular endothelial growth factor receptor-2 (VEGFR-2) [68]. The toxicity profile of these TKIs is generally manageable and has been deemed clinically acceptable. These agents are of particular interest in the treatment of ES-SCLC patients who have exhausted at least two previous chemotherapy regimens [70].

Disordered fibroblast growth factor/FGF receptor (FGF/FGFR) signaling within the TME has been implicated in facilitating immune evasion and catalyzing tumor advancement. Preclinical evidence posits the potential synergistic effect of coupling FGFR inhibitors with ICB for enhanced therapeutic outcomes [71]. TMB, a burgeoning biomarker, has shown predictive utility in the responsiveness to ICB therapies. Elevated TMB levels in patients with EGFR-mutant lung cancer were associated with reduced time to treatment discontinuation (TTD) (HR, 0.46; P = 0.0008) and diminished OS (HR, 0.40; P = 0.006) [72]. Consequently, FGFR alterations may be influential in modulating T-cell infiltration, immune cell activation, and immune checkpoint expression in the TME.

## ICBs combined with radiotherapy

Radiation therapy exerts a dual role: it eliminates tumor cells while concurrently modulating the TME to heighten immune system recognition of neoplastic cells. Meanwhile, the application of Prophylactic Cranial Irradiation could underscore the increasing importance of preventive modalities in mitigating morbidity and mortality. Specifically, radiation therapy augments the expression of tumor-associated antigens, catalyzes cytokine liberation, engenders dendritic cell recruitment, and most saliently, incites the proliferation and initiation of cytotoxic CD8+ T cells within the TME. This sequence of immunologic events selectively activates T cells, culminating in the immunogenic demise of antigen-bearing cancer cells [73]. Particularly noteworthy is the prominence of dendritic cells, crucial facilitators of antigen presentation within the immune system. By modulating this antigenic presentation, dendritic cells not only invigorate immune responses but also manifest minimal toxic and adverse effects, thus potentiating the efficacy of immunotherapeutic interventions.

In the domain of NSCLC, immunotherapeutic strategies are deployed as the front-line treatment for patients with inoperable, locally advanced disease states [74]. However, the combined modality of thoracic radiation therapy (TRT) or brain radiation therapy (BRT) with immunotherapy has not yet been comprehensively validated in the context of LS-SCLC. Therefore, the integration of ICBs and radiation therapy may proffer a novel therapeutic avenue for ES-SCLC, although prognostic indices require further elaboration [75].

In the arena of SCLC, the clinical application of concurrent radiotherapy, chemotherapy, and immunotherapy remains a subject of ongoing research without formalized guidelines. Nonetheless, some preliminary evidence has emerged. For instance, a phase 1/2 trial investigating pembrolizumab alongside concurrent chemoradiation therapy for LS-SCLC has reported encouraging outcomes, with a median progression-free survival of 19.7 months and an overall survival of 39.5 months [76]. Additionally, a phase I trial aimed to evaluate the feasibility and safety of combining pembrolizumab with radiation therapy in patients with ES-SCLC (NCT02402920). The study revealed a median progression-free survival time of 6.1 months and an overall survival time of 8.4 months, further attesting to the regimen's tolerability and minimal high-grade adverse event profile [77].

In a broader context, the therapeutic landscape of NSCLC may offer instructive paradigms. Specifically, two strategies warrant further scrutiny: (1) the concurrent integration of chemotherapy, radiotherapy, and immunotherapy, a combination that has been beneficial in NSCLC and could, theoretically, be translatable to SCLC; and (2) the sequential implementation of chemoradiotherapy followed by immunotherapy, an approach that has shown promise in NSCLC and might serve as a conceptual scaffold for similar explorations in SCLC. In summary, while these lines of inquiry are preliminary, they hint at the potential for evolving a more effective, multimodal therapeutic regimen for SCLC. The accrued data thereby merit rigorous, methodologically sound exploration to substantiate the clinical efficacy and safety of these combined interventions.

The KEYNOTE-799 study and the PACIFIC trial both explore the confluence of immunotherapy with concurrent chemoradiation therapy (cCRT) in the management of unresectable, locally advanced, stage III non-small-cell lung cancer (NSCLC), albeit with variations in their respective protocols and objectives [78–80].

In the KEYNOTE-799 investigation (NCT03631784), patients were randomized into two distinct cohorts. Cohort A was administered carboplatin, paclitaxel, and pembrolizumab in conjunction with standard thoracic radiotherapy. Alternatively, cohort B received cisplatin, pemetrexed, and pembrolizumab along with thoracic radiotherapy. The study reported promising results: progression-free survival at 6 months was 81.4% in cohort A and 85.2% in cohort B. Moreover, the 6-month overall survival rates were 87.2% and 94.8% in cohorts A and B, respectively [80].

Contrastingly, the PACIFIC study (NCT02125461) served as a randomized controlled trial aimed at evaluating consolidation therapy versus placebo following cCRT. Notably, the majority of patients (76.8%) underwent cCRT, while 14.6% received sequential chemoradiotherapy. Durvalumab showcased a median overall survival (OS) of 47.5 months and a median PFS of 16.9 months. The 5-year OS and PFS rates were 42.9% and one-third, respectively. The study also included pertinent data on radiation-induced pneumonia, reporting occurrences in 17.9% of the study participants [78, 79].

In addition, the toxicity of limited stage chemoradiotherapy combined with immunization is great which could impact the efficacy outcomes [81]. Radiation pneumonitis is a notable adverse event associated with thoracic radiotherapy, occurring in an estimated 5–15% of patients undergoing treatment for lung cancer. Importantly, a study underscores the value of dosimetric parameters V (30) and V (40) as crucial metrics for predicting the risk of RP in SCLC patients [82, 83]. Importantly, a study underscores the value of dosimetric parameters V (30) and V (40) as crucial metrics for predicting the risk of RP in SCLC patients [84].

## Bispecific antibodies

In recent years, the advent of bispecific antibodies (BsAbs) has emerged as a transformative stride in the arena of targeted therapeutics. Unlike traditional monoclonal antibodies (MoAbs) which engage with a single antigen, bsAbs are equipped with dual specificity, allowing them to bind to two different antigens or epitopes simultaneously. This capability has elevated their potential role in various medical domains, notably in tumor immunotherapy, where they are hailed for their enhanced therapeutic efficacy [85].

### Anti-PD-1 and anti-CTLA-4 bispecific antibody

Cadizumab (also known as AK 104), a bifunctional antibody targeting both PD-1 and CTLA-4, has received approval from the NMPA in China for the therapeutic management of patients with recurrent or metastatic cervical cancer [86]. In the domain of SCLC, an ongoing clinical investigation (NCT05505825) is being conducted to assess the safety and efficacy of a combinatorial regimen comprising cadizumab (AK 104) and chiauranib in patients diagnosed with ES-SCLC. The primary objectives of this study encompass the quantification of clinical response rates and the systematic evaluation of safety profiles, with particular focus on the incidence and severity of treatment-related adverse events.

### Anti-PD-1 and VEGF bispecific antibody

AK112, a bispecific antibody targeting PD-1 and Vascular Endothelial Growth Factor (VEGF), is currently under investigation in a Phase Ib clinical trial (NCT05116007). The study aims to evaluate the safety and efficacy of AK112 in combination with standard chemotherapy agents—specifically, carboplatin and etoposide—in patients diagnosed with ES-SCLC. The overarching objective of this clinical exploration is to identify novel therapeutic modalities to enhance treatment outcomes in ES-SCLC. The trial is structured in two distinct phases: an induction phase, during which participants will be administered a combination of AK112, carboplatin, and etoposide intravenously over a 12-week period; followed by a maintenance phase where the administration of AK112 will be continued as a monotherapy for up to 24 months.

### Anti-PD-L1 and CD47 bispecific antibody

IMM2520 represents a bispecific antibody targeting both PD-L1 and CD47 and is currently under investigation for its therapeutic potential in patients with advanced solid malignancies. A multi-center Phase I clinical trial is ongoing in China to evaluate not only its safety and tolerability but also its pharmacokinetic properties and anti-neoplastic activity (NCT05780307). The study incorporates both accelerated titration and the conventional "3 + 3" design to ascertain the maximum tolerated dose (MTD) and to establish the recommended dose for Phase II trials during the dose-escalation phase.

### Anti-DLL3 and CD47 bispecific antibody

A Phase I clinical trial (NCT05652686) is currently underway to assess PT217, a bispecific antibody targeting human Delta-like ligand 3 (huDLL3) and human CD47 (huCD47), in patients with advanced or refractory malignancies that express DLL3. The primary objectives of this trial include the evaluation of safety, tolerability, pharmacokinetic parameters, and preliminary therapeutic efficacy of PT217.

### Anti-DLL3 and CD3 bispecific antibody

A Phase I trial of the DLL3/CD3 bispecific T-cell engager BI 764532 in DLL3-positive small-cell lung cancer and neuroendocrine carcinomas (NCT04429087). This paper delineates the findings of a Phase I clinical trial focused on the evaluation of a bispecific T-cell engager, designated as BI 764532, in patients with SCLC and additional neuroendocrine carcinomas. The primary objectives of this investigation are to ascertain the MTD of BI 764532 and to conduct a comprehensive analysis of its safety, tolerability, pharmacokinetics, and preliminary antitumor efficacy when employed as monotherapy. DLL3 has emerged as a critical therapeutic target owing to its aberrant expression on these malignancies. The manuscript elaborates on the study's design and potential ramifications for augmenting the clinical outcomes in patients with SCLC and neuroendocrine carcinomas [87]. As of December 28, 2022, a total of 90 patients had been administered a singular dose of BI 764532, with 47 patients diagnosed with SCLC, 13 of whom are still under active treatment. A notable 53% of the study population had previously been treated with PD-1/PD-L1 inhibitors, while 77% had undergone at least two prior therapeutic regimens. The median duration of treatment was observed to be 43 days. An analysis of tumor response for 34 heavily pretreated SCLC patients, encompassing all treatment modalities and dose levels, revealed an overall response rate of 33% (all partial responses) among those (n = 24) who received the target dose of BI 764532. The disease control rate was established at 66%. BI 764532 exhibited clinically manageable tolerability profiles and met the criteria for MTD. Promising therapeutic efficacy was observed among the cohort of SCLC patients who had undergone extensive pretreatment [88].

Agents such as AK104 IV and cadonilimab are predominantly deployed in the treatment of ES-SCLC, whereas XmAb20717 is under investigation for its utility across both primary subtypes of SCLC. A notable phase II clinical study is investigating the efficacy and safety of JS201, a bispecific antibody targeting both PD-1 and TGF-β, when used in combination with the kinase inhibitor lenvatinib for SCLC refractory to prior chemotherapy and PD-L1 inhibition. On the other hand, AK112, which targets both PD-1 and VEGF, is in a phase I trial in conjunction with traditional chemotherapy for patients with ES-SCLC. Two other agents, M7824 (anti-PD-L1/TGF-β) and PM8002 (anti-PD-L1/VEGF), are also in trials where they are integrated with chemotherapy. Specifically, M7824 is combined primarily with topotecan or temozolomide for relapsed SCLC, while PM8002 is co-administered with paclitaxel as a second-line treatment strategy.

## ACT

ACT involves the isolation, manipulation, and subsequent re-infusion of autologous immune cells back into the patient. T lymphocytes can be genetically engineered to target malignant cells that express specific tumor-associated antigens, including strategies utilizing chimeric antigen receptor T cells (CAR-T) and T-cell receptor-engineered T cells (TCR-T). While the modified TCR is engineered to have a high-affinity interaction with specific major histocompatibility complex (MHC) peptide epitope complexes, CAR-T cells function independently of MHC and incorporate single-chain variable fragments (scFvs) with specificity for tumor-associated antigens.

### CAR-T

Emerging therapeutic targets under investigation in early-stage clinical trials encompass Mesothelin (MSLN), Membrane-Associated Glycoprotein (MUC-1), Carcinoembryonic Antigen (CEA), Glypican-3 (GPC3), Human Epidermal Growth Factor Receptor 2 (HER2), and Receptor Tyrosine Kinase-Like Orphan Receptor 1 (ROR1) [89, 90]. MSLN serves as a tumor differentiation antigen predominantly localized to mesothelial cells lining the pleura, peritoneum, and pericardium [91, 92]. It is aberrantly overexpressed in a myriad of human malignancies, including mesothelioma, ovarian cancer, pancreatic adenocarcinoma, lung adenocarcinoma, and cholangiocarcinoma [92–94]. CEA functions as both a prognostic and predictive biomarker, specifically in the context of lung cancer, where it has utility in gauging overall survival [95]. HER2 is considered an adverse prognostic factor in various neoplasms, most notably in SCLC and early-stage NSCLC [96]. ROR1, a member of the ROR receptor family localized to the cell membrane, is the subject of investigations into its putative role in cancer cell metastasis [97].

### TCR-T

TCR therapy uses the innate mechanism of T cells to target tumor antigens. Through genetically modifying isolated T cells, the ex vivo cells express the cancer antigen-specific T cell receptor (TCR) generated by TCR engineering of patient isolated T cells (TCRT) [98]. TCRs can only recognize specific oncogenic peptides presented by human leukocyte antigen (HLA) class I on tumor cells or APC surfaces [99]. A phase I/II trial of NY-ESO-1-specific TCR-T treatment for synovial sarcoma and melanoma showed that 61% Synovial cell sarcoma and 55% melanoma had therapeutic responses (NCT03029273). Another clinical trial is currently investigating the safety and dose of TCR Gene therapy targeting KK-LC-1 lung cancer patients (NCT05035407). In recent years, TCR-T therapy has made a breakthrough in many tumors. However, there are few safe and effective targets due to the possible off-target, dose-limiting cytotoxicity, autoimmune toxicity, and cytokine-related toxicity [100]. Therefore, TCR-T therapy would be widely used clinically, these challenges must be addressed.

### Vaccine

Cancer vaccines, designed to exploit tumor-associated antigens to stimulate host immune responses against malignancies, have demonstrated promise, particularly in lung cancer contexts [90]. INGN-225, an adenoviral vaccine expressing a modified p53 antigen targeting dendritic cells, elicited a specific anti-p53 immune response in 41% of patients with SCLC, highlighting its potential therapeutic relevance given the frequent mutations in the p53 gene [101]. Separately, a DNA-based neoantigen vaccine platform is under investigation for personalized tumor immunotherapy. In murine models, this platform effectively inhibited melanoma growth and attenuated lung metastases, corroborating the prospective utility of neoantigen-based vaccines in personalized approaches and serving as a prevalidation for effective neoantigen selection [102]. Antibody–drug conjugates (ADCs), such as brentuximab vedotin targeting CD30-positive malignancies, offer another avenue for cancer therapy. The cytotoxic payload of brentuximab vedotin, derived from the dolastatin family of microtubule inhibitors, not only exerts direct antitumor effects but also facilitates dendritic cell maturation, antigen uptake, and migration to lymph nodes, thereby augmenting T-cell activation. Combination regimens involving dolastatins and tumor antigen-specific vaccines or immune checkpoint inhibitors demonstrate significant therapeutic synergies [103]. Collectively, these findings underscore the viability of vaccine-based therapies in the evolving landscape of SCLC treatment.

## Oncolytic virus

Oncolytic viruses, highly replicative entities with a proclivity for selectively infecting and lysing neoplastic cells both in vitro and in vivo, have garnered increasing attention in the realm of cancer therapeutics [104, 105]. These agents exert their antitumor effects through a multitude of mechanisms, ranging from direct cellular lysis to induction of apoptosis and potentiation of immune cell-mediated cytotoxicity. Recent evidence suggests that oncolytic viruses can also amplify the host's antitumor immune response, thereby enhancing the efficacy of immunotherapeutic regimens. There is burgeoning interest in combining oncolytic virotherapy with ICBs to broaden the therapeutic landscape for solid tumor [105]. In the context of SCLC, a genetically engineered myxoma virus (MYXV) demonstrated robust tumor-specific cytotoxicity in murine models and elicited immune cell infiltration into the TME [106]. Consequently, oncolytic virotherapy is emerging as a highly promising modality, with the potential to serve as a seminal advancement in cancer therapeutics, particularly following the success of ICBs in immunotherapy [107].

## Monotherapy

### DLL3

Delta-like ligand 3 (DLL3), an inhibitory ligand in the Notch signaling pathway, is characteristically overexpressed in high-grade neuroendocrine malignancies, such as SCLC. The expression of DLL3, observed in over 80% of SCLC, is associated with the progression of these tumors. The distinct expression pattern of DLL3 in SCLC, as opposed to its minimal or absent expression in normal tissues, highlights its potential as a therapeutic target in SCLC treatment [108]. Furthermore, while not intimately associated with patients' physiological characteristics or tumorigenesis, DLL3 has been identified as a predictive biomarker [89]. Therapeutic interventions targeting DLL3 span a wide array of modalities including T-cell engager (TCE) molecules—monospecific, bispecific, and trispecific antibodies, ADCs such as Rova-T, CAR-T such as AMG119, and CAR-NK cell therapies [109].

A Phase 1 clinical trial (NCT05680922) is underway to investigate DLL3-targeted CAR-T cells in patients with extensive-stage SCLC and large-cell neuroendocrine lung cancer. The study design incorporates both dose-escalation and cohort-expansion phases to assess safety and efficacy. Rovalpituzumab tesirine (Rova-T) is an ADC that consists of a humanized monoclonal antibody specifically targeting DLL3 (SC16), conjugated to a membrane-permeable pyrrolobenzodiazepine (PBD) dimer toxin, serving as the warhead. This conjugation employs a lysosome-targeted, protease-sensitive dipeptide linker, designed for enhanced precision in delivering the cytotoxic agent [110, 111]. Rova-T has been administered to over 1000 patients, both as a standalone treatment and in combination with various chemotherapy and immunotherapy regimens, across more than 10 clinical trials, including two pivotal phase 3 studies. The initial human study (FIH) of Rova-T demonstrated an ORR of 18%, which notably increased to 38% among patients with high DLL3 expression in SCLC (defined as DLL3 expression in ≥ 50% of tumor cells as determined by IHC) [111]. Additionally, a separate Phase 1 multicenter trial in China (NCT05507593) aims to evaluate the safety and therapeutic potential of DLL3-CAR-NK cells in treating relapsed and refractory ES-SCLC.

Tarlatamab, a First-in-Class DLL3-Targeted Bispecific T-Cell Engager, in Recurrent Small-Cell Lung Cancer: An Open-Label, Phase I Study (NCT03319940).

This investigation assessed the safety and therapeutic efficacy of tarlatamab in patients afflicted with recurrent SCLC. The study encompassed a total of 107 patients, administered varying dosages of tarlatamab, and was structured around two primary endpoints: safety profile and antitumor activity, inclusive of overall survival metrics. The observed objective response rate was 23.4%, accompanied by a median duration of response equating to 12.3 months. The disease control rate was ascertained to be 51.4%, while median progression-free survival and overall survival were recorded at 3.7 months and 8.6 months, respectively. These data collectively intimate that tarlatamab holds promise as a potentially efficacious treatment regimen for recurrent SCLC, warranting further investigational studies [112].

HPN328, a cutting-edge DLL3-targeting Tri-specific T cell Activating Construct (TriTAC), is currently under evaluation in a Phase 1/2 clinical trial. This trial is focused on enrolling patients with advanced cancers characterized by DLL3 expression, encompassing SCLC and various other neuroendocrine malignancies (NCT04471727). HPN328, targeting DLL3, has shown promise in treating relapsed/refractory SCLC as a solo therapy. To augment its efficacy, the study investigated its combination with immunotherapies, utilizing a novel human CD3ε (hCD3ε) immunocompetent mouse model. This model, responsive to TriTAC molecules, validated HPN328’s potential when tested with an EGFR-targeting TriTAC. In these mice, HPN328 not only inhibited DLL3-expressing MC38 tumor growth but also increased PD-L1 expression, indicating enhanced therapeutic potential. Combining HPN328 with an anti-PD-L1 antibody resulted in significantly improved anti-tumor activity. This synergy underscores the feasibility of HPN328 with anti-PD-L1 antibodies, like atezolizumab and durvalumab, currently used in first-line SCLC therapy. These findings pave the way for clinical trials combining HPN328 with atezolizumab, representing a potential advancement in SCLC treatment strategies [113].

### TIGIT

T-cell immunoreceptor with immunoglobulin and TIGIT is a prominent immune checkpoint receptor expressed on the surface of natural killer (NK) cells, effector T cells, and regulatory T cells (Tregs). Its ligands, CD112 and CD155, are not only found on tumor cells but also on antigen-presenting cells (APCs) and T cells [114]. Experimental evidence suggests that blockade of TIGIT mitigates NK cell exhaustion and augments NK cell-mediated antitumor immunity in several murine tumor models [115]. Furthermore, the binding of TIGIT to its ligands culminates in the inhibition of interleukin-12 (IL-12) production, thereby perturbing the homeostatic architecture of T-cell populations [116]. In the TME, TIGIT is frequently co-expressed on tumor-infiltrating lymphocytes (TILs), particularly in concert with other immune checkpoint molecules such as PD-1, lymphocyte activation gene-3 (LAG-3), and T-cell immunoglobulin and mucin-domain containing-3 (TIM-3). This co-expression appears to compromise the functionality of effector T cells, impeding their antitumor efficacy [117].

Presently, a Phase I clinical trial (NCT03119428) is underway to explore the therapeutic utility of TIGIT in locally advanced and metastatic solid tumors. The trial aims to delineate whether TIGIT functions optimally when combined with other immune checkpoint inhibitors or co-inhibitory/co-stimulatory signaling antibodies; these interactions remain the subject of ongoing investigation [118].

A Phase III clinical trial (NCT04256421) has been instituted to investigate the therapeutic efficacy of tiragolumab in combination with atezolizumab, carboplatin, and etoposide in chemotherapy-naive patients with ES-SCLC. Study participants will be stratified into two distinct treatment arms: the first will receive tiragolumab concomitant with atezolizumab, carboplatin, and etoposide, while the second arm will be administered a placebo in addition to atezolizumab, carboplatin, and etoposide. The principal objective of the study is to comparatively evaluate the efficaciousness of these regimen combinations during the induction phase, followed by a maintenance phase utilizing either atezolizumab with tiragolumab or atezolizumab with a placebo.

Separately, a Phase II clinical trial sponsored by BeiGene (NCT04952597) is underway to explore the synergistic potential of ociperlimab and tislelizumab when administered with concurrent chemoradiotherapy in patients with untreated LS-SCLC. Given that LS-SCLC is a condition characterized by substantial unmet clinical needs, the utilization of a combination of anti-TIGIT antibody and anti-PD-1/L1 antibody could potentially amplify the immunotherapeutic effect while maintaining a tolerable safety profile. This trial aims to provide a preliminary evaluation of the efficacy and safety of this combinatorial approach.

## Precision medicine

SCLC exhibits a marked degree of genetic heterogeneity, a finding that complexifies its therapeutic landscape. While a litany of seminal studies have delineated somatic mutations targeting canonical oncogenes and tumor suppressor genes, the diversity in genetic aberrations in SCLC adds another layer of intricacy to its molecular profile. This inherent genetic complexity necessitates more nuanced approaches for both targeted and systemic therapies [119]. Given its aggressive nature, SCLC demands personalized and precisely targeted therapeutic strategies [33]. In pursuit of targeted therapy for SCLC, various avenues of research have been explored [119]. In recent years, precision medicine has emerged as a prominent and dynamic field, witnessing significant transformations in personalized treatment approaches. Nevertheless, research pertaining to targeted therapies in SCLC remains comparatively constrained, particularly when addressing precision medicine strategies following initial drug resistance. Consequently, there exists a compelling need for further investigations into targeted treatments for SCLC. These endeavors aim to craft more tailored interventions for patients, ultimately extending their life expectancy.

## Current status and future directions

ASCL1 serves a critical role in the neuroendocrine differentiation and subsequent carcinogenesis of SCLC, thereby presenting itself as a potential focal point for future research endeavors [120]. ICB therapies occupy a preeminent position as the predominant form of immunotherapy, demonstrating vast potential for therapeutic advancement in SCLC management. The extant literature, underscoring the imperative for combining various therapeutic modalities in the management of SCLC. These include ICBs adjoined with chemotherapy, radiotherapy, targeted therapy, as well as other immunotherapeutic modalities. Notably, given the complementary functionalities of immune checkpoint pathways, specifically CTLA-4 and PD-1/PD-L1, synergistic effects may be realized when co-administering different ICBs. Moreover, the confluence of chemoradiotherapy with immunotherapy holds promise in potentiation of antitumor immune responses. Specifically, chemoradiotherapy may precipitate rapid tumor lysis and consequent antigen release, thereby augmenting tumor cell immunogenicity [121].

Both immunotherapy and chemo-immunotherapy represent pivotal approaches in the management of SCLC. It must be underscored that SCLC exhibits the most dire prognosis among lung malignancies; a significant fraction of patients receiving initial chemotherapy regimens experience disease relapse within a year, marked by abysmal survival outcomes. ICBs has radically disrupted a three-decade-long therapeutic stalemate in SCLC management, thus accentuating the imperative for continued investigative efforts aimed at optimizing the clinical utility of ICBs in this malignancy [51]. At present, FDA has conferred approval for nivolumab and pembrolizumab as PD-1 inhibitors in the clinical management of neoplastic disorders. Concurrently, atezolizumab and durvalumab have received regulatory endorsement as PD-L1 antagonistic antibodies for oncologic applications [7]. In comparative analyses, the amalgamation of immune checkpoint inhibitors with chemotherapy demonstrates a propitious inclination toward enhancing both OS and PFS metrics. Consequently, this combinatory therapeutic approach is progressively crystallizing as the putative first-line regimen for the management of ES-SCLC [122]. Nonetheless, it is imperative to note that existing regimens incorporating the confluence of immunotherapy and chemotherapy are predominantly focused on ES-SCLC, leaving a lacuna in real-world data. Moreover, in a specific cohort of patients with LS-SCLC, the utilization of immunotherapy as a component of the initial systemic therapy regimen did not manifest a statistically significant enhancement in OS [123].

A further challenge in the therapeutic landscape for SCLC resides in the dual concerns of limited treatment efficacy and associated adverse events. TME is a dynamic and complex ecosystem that plays a role in tumorigenesis and metastasis [51]. Innate immune cells, acquired immune cells, and tumor cells are the major components in the TME [124]. Immune cells are crucial in tumorigenesis. Tumor-associated immune cells are involved in inhibiting tumor growth and promoting tumor progression. Tumor immune cells destroy cancer cells in the early stages of tumorigenesis, but cancer cells can promote tumorigenesis by suppressing the cytotoxic effects of immune cells [125–127]. In a randomized phase II study, it assess the efficacy of ICB with or without radiation in relapsed SCLC. The most frequent adverse events in the study were fatigue, pain, diarrhea, elevated amylase, thrombocytopenia and dyspnea [128]. Although immunotherapy has made great progress in the treatment of SCLC patients, there have been limited improvements in survival rates for SCLC patients with the current immune checkpoint inhibitors PD-1/PD-L1 and CTLA-4 [129]. Through preclinical analyses, SCLC have been classified into numerous subtypes with specific characteristics, but so far there are no existing clinical subtype distinction which may contribute to negative clinical trial outcomes [130]. It is difficult to predict who will respond to ICBs, thus there remains a need to discover new predictive immunotherapy biomarkers. Furthermore, because the lack of response and disease progression after an initial progress, resistance to ICBs in SCLC is frequent [131]. Six types of potential treatable signaling pathways in SCLC has been discovered, but there is still a lack of understanding of their roles in SCLC tumor biology and the promotion of cancer growth [132]. Given these obstacles, the imperative for robust preventative measures and vigilant early diagnosis cannot be overstated [60].

Since the turn of the last decade, substantial advancements have been realized in the overall survival rates for patients diagnosed with LS-SCLC. These improvements are primarily attributable to the integration of dose—hyperfractionated thoracic radiotherapy and prophylactic cranial irradiation alongside conventional systemic chemotherapy regimens [133]. Notably, for the first time in over three decades, studies have elucidated that the strategic amalgamation of immunotherapy with chemotherapy augments survival outcomes for patients with extensive-stage SCLC, thereby crystallizing a novel standard-of-care for frontline therapeutic interventions [134]. Yet, it is imperative to optimize the efficaciousness of immunotherapy in the SCLC therapeutic landscape and to delineate patient subsets most likely to derive benefit from these innovative treatments. Accordingly, burgeoning research efforts are concentrated on identifying predictive biomarkers for immunotherapy in SCLC and devising strategies to bolster its efficacy and survival rates.

In summary, the prognostic landscape for SCLC treatment is optimistic. The amalgamation of immunotherapy with other modalities is poised to occupy a linchpin role in future treatment paradigms. Multidisciplinary research endeavors are likely to unveil increasingly efficacious treatment strategies, thereby incrementally augmenting the survival rates of patients with SCLC. Concurrently, advancements in individualized precision medicine stand to offer patients improved treatment outcomes, thereby enhancing the overall therapeutic index for this malignancy.

## Conclusions

In recent years, the domain of immunotherapy for the treatment of SCLC has experienced an accelerated evolution, accompanied by significant advancements. A burgeoning volume of studies is being dedicated to the analysis of clinical trials and the evaluation of novel therapeutic agents. Immune checkpoint inhibitors continue to hold a preeminent position in the SCLC immunotherapeutic armamentarium. Notably, the synergistic integration of multiple treatment modalities emerges as pivotal in enhancing patient survival outcomes. Additionally, other therapeutic paradigms that have demonstrated success in diverse oncological contexts are under rigorous investigation. The prospects for the introduction of more efficacious treatment regimens appear promising. SCLC is distinguished by high recurrence rates, small beneficiary populations, and low survival benefits. It is necessary to explore new targets for key molecules and signals in SCLC and the development of drugs with novel mechanisms in immunotherapy to increase patient survival time. Concomitantly, the trajectory toward individualized precision medicine stands as a promising future direction with a rapid development of technology. Precision medicine brings a glimmer of hope for SCLC patients.

## Data Availability

No datasets were generated or analysed during the current study.

## References

[1] Sung H, et al. Global cancer statistics 2020: GLOBOCAN estimates of incidence and mortality worldwide for 36 cancers in 185 countries. CA-A Cancer J Clin. 2021;71(3):209–49. 10.3322/caac.21660 33538338

[2] Khanmohammadi A, et al. Electrochemical biosensors for the detection of lung cancer biomarkers: a review. Talanta. 2020;206: 120251. 31514848 10.1016/j.talanta.2019.120251

[3] Horn L, Reck M, Spigel DR. The future of immunotherapy in the treatment of small cell lung cancer. Oncologist. 2016;21(8):910–21. 27354668 10.1634/theoncologist.2015-0523 PMC4978554

[4] Kalemkerian GP, et al. Small cell lung cancer. J Natl Compr Canc Netw. 2013;11(1):78–98. 23307984 10.6004/jnccn.2013.0011 PMC3715060

[5] Zhang S, Liu J, Cheng Y. Clinical development of immune checkpoint inhibitors in patients with small cell lung cancer. Chin J Lung Cancer. 2017;20(9):623–8. 10.3779/j.issn.1009-3419.2017.09.06 PMC5973375 28935016

[6] Morabito A, Rolfo C. Small cell lung cancer: a new era is beginning? Cancers. 2021;13(11):2646. 34071158 10.3390/cancers13112646 PMC8197965

[7] Yang Y, Yu Y, Lu S. Effectiveness of PD-1/PD-L1 inhibitors in the treatment of lung cancer: brightness and challenge. Sci China Life Sci. 2020;63(10):1499–514. 32303964 10.1007/s11427-019-1622-5

[8] Lim JU, Kang HS. A narrative review of current and potential prognostic biomarkers for immunotherapy in small-cell lung cancer. Ann Transl Med. 2021;9(9):809. 34268422 10.21037/atm-21-68 PMC8246157

[9] Wang Q, et al. Research progress of immunotherapy and prognostic markers in small cell lung cancer. Zhongguo Fei Ai Za Zhi. 2020;23(3):182–8. 32102135 10.3779/j.issn.1009-3419.2020.03.08 PMC7118334

[10] Rijavec E, et al. Current state of the art and future perspectives with immunotherapy in the management of small cell lung cancer. Expert Rev Respir Med. 2021;15(11):1427–35. 34590937 10.1080/17476348.2021.1987887

[11] Yang S, Zhang Z, Wang Q. Emerging therapies for small cell lung cancer. J Hematol Oncol. 2019;12(1):47. 31046803 10.1186/s13045-019-0736-3 PMC6498593

[12] Liu SV, et al. Updated overall survival and PD-L1 subgroup analysis of patients with extensive-stage small-cell lung cancer treated with atezolizumab, carboplatin, and etoposide (IMpower133). J Clin Oncol. 2021;39(6):619–30. 33439693 10.1200/JCO.20.01055 PMC8078320

[13] Tsiouprou I, Zaharias A, Spyratos D. The role of immunotherapy in extensive stage small-cell lung cancer: a review of the literature. Can Respir J. 2019;2019:6860432. 31781314 10.1155/2019/6860432 PMC6875088

[14] Iams WT, Porter J, Horn L. Immunotherapeutic approaches for small-cell lung cancer. Nat Rev Clin Oncol. 2020;17(5):300–12. 32055013 10.1038/s41571-019-0316-z PMC7212527

[15] Paz-Ares L, et al. Durvalumab plus platinum-etoposide versus platinum-etoposide in first-line treatment of extensive-stage small-cell lung cancer (CASPIAN): a randomised, controlled, open-label, phase 3 trial. Lancet. 2019;394(10212):1929–39. 31590988 10.1016/S0140-6736(19)32222-6

[16] Konala VM, et al. Use of immunotherapy in extensive-stage small cell lung cancer. Oncology. 2020;98(11):749–54. 32663833 10.1159/000508516

[17] Lai Y, et al. Emerging trends and new developments in monoclonal antibodies: a scientometric analysis (1980–2016). Hum Vaccin Immunother. 2017;13(6):1–10. 28301271 10.1080/21645515.2017.1286433 PMC5489293

[18] Chen C, et al. Emerging trends in regenerative medicine: a scientometric analysis in CiteSpace. Expert Opin Biol Ther. 2012;12(5):593–608. 22443895 10.1517/14712598.2012.674507

[19] Raso MG, Bota-Rabassedas N, Wistuba II. Pathology and classification of SCLC. Cancers. 2021;13(4):820. 33669241 10.3390/cancers13040820 PMC7919820

[20] Liu J, et al. Genomic features of Chinese small cell lung cancer. BMC Med Genomics. 2022;15(1):117. 35596192 10.1186/s12920-022-01255-3 PMC9123817

[21] Topalian SL, Drake CG, Pardoll DM. Immune checkpoint blockade: a common denominator approach to cancer therapy. Cancer Cell. 2015;27(4):450–61. 25858804 10.1016/j.ccell.2015.03.001 PMC4400238

[22] Ribas A, Wolchok JD. Cancer immunotherapy using checkpoint blockade. Science. 2018;359(6382):1350. 29567705 10.1126/science.aar4060 PMC7391259

[23] Mortezaee K. Immune escape: a critical hallmark in solid tumors. Life Sci. 2020;258: 118110. 32698074 10.1016/j.lfs.2020.118110

[24] Alegre ML, Frauwirth KA, Thompson CB. T-cell regulation by CD28 and CTLA-4. Nat Rev Immunol. 2001;1(3):220–8. 11905831 10.1038/35105024

[25] Black M, et al. Activation of the PD-1/PD-L1 immune checkpoint confers tumor cell chemoresistance associated with increased metastasis. Oncotarget. 2016;7(9):10557–67. 26859684 10.18632/oncotarget.7235 PMC4891140

[26] Zabeti Touchaei A, Vahidi S. MicroRNAs as regulators of immune checkpoints in cancer immunotherapy: targeting PD-1/PD-L1 and CTLA-4 pathways. Cancer Cell Int. 2024;24(1):102. 38462628 10.1186/s12935-024-03293-6 PMC10926683

[27] Hofmann L, et al. Cutaneous, gastrointestinal, hepatic, endocrine, and renal side-effects of anti-PD-1 therapy. Eur J Cancer. 2016;60:190–209. 27085692 10.1016/j.ejca.2016.02.025

[28] Akinleye A, Rasool Z. Immune checkpoint inhibitors of PD-L1 as cancer therapeutics. J Hematol Oncol. 2019. 10.1186/s13045-019-0779-5. 31488176 10.1186/s13045-019-0779-5 PMC6729004

[29] Calles A, et al. The role of immunotherapy in small cell lung cancer. Clin Transl Oncol. 2019;21(8):961–76. 30637710 10.1007/s12094-018-02011-9

[30] El Sayed R, Blais N. Immunotherapy in extensive-stage small cell lung cancer. Curr Oncol. 2021;28(5):4093–108. 34677265 10.3390/curroncol28050347 PMC8534845

[31] Melosky B, et al. Prolonging survival: the role of immune checkpoint inhibitors in the treatment of extensive-stage small cell lung cancer. Oncologist. 2020;25(11):981–92. 32860288 10.1634/theoncologist.2020-0193 PMC7648366

[32] Oser MG, et al. Transformation from non-small-cell lung cancer to small-cell lung cancer: molecular drivers and cells of origin. Lancet Oncol. 2015;16(4):E165–72. 25846096 10.1016/S1470-2045(14)71180-5 PMC4470698

[33] Lantuejoul S, et al. PD-L1 testing for lung cancer in 2019: perspective from the IASLC pathology committee. J Thorac Oncol. 2020;15(4):499–519. 31870882 10.1016/j.jtho.2019.12.107

[34] Planchard D, Le Pechoux C. Small cell lung cancer: new clinical recommendations and current status of biomarker assessment. Eur J Cancer. 2011;47:S272–83. 21943984 10.1016/S0959-8049(11)70173-3

[35] Fan Y, et al. Camrelizumab plus apatinib in extensive-stage SCLC (PASSION): a multicenter, two-stage, phase 2 trial. J Thorac Oncol. 2021;16(2):299–309. 33166719 10.1016/j.jtho.2020.10.002

[36] Hellmann MD, et al. Tumor mutational burden and efficacy of nivolumab monotherapy and in combination with ipilimumab in small-cell lung cancer. Cancer Cell. 2018;33(5):853-861.e4. 29731394 10.1016/j.ccell.2018.04.001 PMC6750707

[37] Karachaliou N, et al. Cellular and molecular biology of small cell lung cancer: an overview. Trans Lung Cancer Res. 2016;5(1):2–15. 10.3978/j.issn.2218-6751.2016.01.02 PMC4758976 26958489

[38] Rossi A, et al. Carboplatin- or cisplatin-based chemotherapy in first-line treatment of small-cell lung cancer: the COCIS meta-analysis of individual patient data. J Clin Oncol. 2012;30(14):1692–8. 22473169 10.1200/JCO.2011.40.4905

[39] Sethi T, et al. Extracellular matrix proteins protect small cell lung cancer cells against apoptosis: a mechanism for small cell lung cancer growth and drug resistance in vivo. Nat Med. 1999;5(6):662–8. 10371505 10.1038/9511

[40] Alagheband Y, et al. Design and fabrication of a dual-drug loaded nano-platform for synergistic anticancer and cytotoxicity effects on the expression of leptin in lung cancer treatment. J Drug Deliv Sci Technol. 2022;73: 103389. 10.1016/j.jddst.2022.103389

[41] Samadzadeh S, et al. In vitro anticancer efficacy of Metformin-loaded PLGA nanofibers towards the post-surgical therapy of lung cancer. J Drug Deliv Sci Technol. 2021;61: 102318. 10.1016/j.jddst.2020.102318

[42] Adlravan E, et al. Potential activity of free and PLGA/PEG nanoencapsulated *Nasturtium officinale* extract in inducing cytotoxicity and apoptosis in human lung carcinoma A549 cells. J Drug Deliv Sci Technol. 2021;61: 102256. 10.1016/j.jddst.2020.102256

[43] Mogheri F, et al. Co-delivery of metformin and silibinin in dual-drug loaded nanoparticles synergistically improves chemotherapy in human non-small cell lung cancer A549 cells. J Drug Deliv Sci Technol. 2021;66: 102752. 10.1016/j.jddst.2021.102752

[44] Salmani Javan E, et al. Development of a magnetic nanostructure for co-delivery of metformin and silibinin on growth of lung cancer cells: possible action through leptin gene and its receptor regulation. Asian Pac J Cancer Prev. 2022;23(2):519–27. 35225464 10.31557/APJCP.2022.23.2.519 PMC9272620

[45] Dadashpour M, et al. Biomimetic synthesis of silver nanoparticles using *Matricaria chamomilla* extract and their potential anticancer activity against human lung cancer cells. Mater Sci Eng C Mater Biol Appl. 2018;92:902–12. 30184820 10.1016/j.msec.2018.07.053

[46] Mellatyar H, et al. 17-DMAG-loaded nanofibrous scaffold for effective growth inhibition of lung cancer cells through targeting HSP90 gene expression. Biomed Pharmacother. 2018;105:1026–32. 30021337 10.1016/j.biopha.2018.06.083

[47] Goldman JW, et al. Durvalumab, with or without tremelimumab, plus platinum-etoposide versus platinum-etoposide alone in first-line treatment of extensive-stage small-cell lung cancer (CASPIAN): updated results from a randomised, controlled, open-label, phase 3 trial. Lancet Oncol. 2021;22(1):51–65. 33285097 10.1016/S1470-2045(20)30539-8

[48] Kaech SM, Cui WG. Transcriptional control of effector and memory CD8(+) T cell differentiation. Nat Rev Immunol. 2012;12(11):749–61. 23080391 10.1038/nri3307 PMC4137483

[49] Garon EB, et al. Five-year long-term overall survival for patients with advanced NSCLC treated with pembrolizumab: results from KEYNOTE-001. J Clin Oncol. 2019;37(18): LBA9015. 10.1200/JCO.2019.37.18_suppl.LBA9015 PMC6768611 31154919

[50] Paz-Ares L, et al. Pembrolizumab plus chemotherapy for squamous non-small-cell lung cancer. N Engl J Med. 2018;379(21):2040–51. 30280635 10.1056/NEJMoa1810865

[51] Li L, et al. Advances in immune checkpoint inhibitors therapy for small cell lung cancer. Cancer Med. 2023;12:11097–106. 36880420 10.1002/cam4.5659 PMC10242320

[52] Fritz H, et al. Polysaccharide K and *Coriolus versicolor* extracts for lung cancer: a systematic review. Integr Cancer Ther. 2015;14(3):201–11. 25784670 10.1177/1534735415572883

[53] Sen T, Gay CM, Byers LA. Targeting DNA damage repair in small cell lung cancer and the biomarker landscape. Transl Lung Cancer Res. 2018;7(1):50–68. 29535912 10.21037/tlcr.2018.02.03 PMC5835589

[54] Knelson EH, Patel SA, Sands JM. PARP inhibitors in small-cell lung cancer: rational combinations to improve responses. Cancers. 2021;13(4):727. 33578789 10.3390/cancers13040727 PMC7916546

[55] Cheng Y, et al. Effect of first-line serplulimab vs placebo added to chemotherapy on survival in patients with extensive-stage small cell lung cancer: the ASTRUM-005 randomized clinical trial. JAMA. 2022;328(12):1223–32. 36166026 10.1001/jama.2022.16464 PMC9516323

[56] Wang J, et al. Adebrelimab or placebo plus carboplatin and etoposide as first-line treatment for extensive-stage small-cell lung cancer (CAPSTONE-1): a multicentre, randomised, double-blind, placebo-controlled, phase 3 trial. Lancet Oncol. 2022;23(6):739–47. 35576956 10.1016/S1470-2045(22)00224-8

[57] Horn L, et al. First-line atezolizumab plus chemotherapy in extensive-stage small-cell lung cancer. N Engl J Med. 2018;379(23):2220–9. 30280641 10.1056/NEJMoa1809064

[58] Rudin CM, et al. Pembrolizumab or placebo plus etoposide and platinum as first-line therapy for extensive-stage small-cell lung cancer: randomized, double-blind, phase III KEYNOTE-604 Study. J Clin Oncol. 2020;38(21):2369–79. 32468956 10.1200/JCO.20.00793 PMC7474472

[59] Owonikoko TK, et al. Nivolumab and ipilimumab as maintenance therapy in extensive-disease small-cell lung cancer: CheckMate 451. J Clin Oncol. 2021;39(12):1349–59. 33683919 10.1200/JCO.20.02212 PMC8078251

[60] Yu L, et al. Opportunities and obstacles of targeted therapy and immunotherapy in small cell lung cancer. J Drug Target. 2021;29(1):1–11. 32700566 10.1080/1061186X.2020.1797050

[61] Schally AV, Nagy A. Cancer chemotherapy based on targeting of cytotoxic peptide conjugates to their receptors on tumors. Eur J Endocrinol. 1999;141(1):1–14. 10407215 10.1530/eje.0.1410001

[62] Schally AV. New approaches to the therapy of various tumors based on peptide analogues. Horm Metab Res. 2008;40(5):315–22. 18491250 10.1055/s-2008-1073142

[63] Sen T, et al. Targeting DNA damage response promotes antitumor immunity through STING-mediated T-cell activation in small cell lung cancer. Cancer Discov. 2019;9(5):646–61. 30777870 10.1158/2159-8290.CD-18-1020 PMC6563834

[64] Li D, et al. Efficacy and safety of fluzoparib combined with anlotinib in extensive stage small cell lung cancer after first-line platinum-based chemotherapy: a multi-center, single-arm prospective phase II clinical study (STAMP study). BMC Cancer. 2023;23(1):753. 37580661 10.1186/s12885-023-11230-5 PMC10424452

[65] Kong T, et al. Anlotinib plus etoposide and cisplatin/carboplatin as first-line therapy for extensive-stage small cell lung cancer (ES-SCLC): a single-arm, phase II study. Invest New Drugs. 2022;40(5):1095–105. 35788937 10.1007/s10637-022-01279-7

[66] Cheng Y, et al. Tislelizumab plus platinum and etoposide versus placebo plus platinum and etoposide as first-line treatment for extensive-stage SCLC (RATIONALE-312): a multicenter, double-blind, placebo-controlled, randomized, phase 3 clinical trial. J Thorac Oncol. 2024. 10.1016/j.jtho.2024.03.008. 38460751 10.1016/j.jtho.2024.03.008

[67] Cheng Y, et al. Anlotinib vs placebo as third- or further-line treatment for patients with small cell lung cancer: a randomised, double-blind, placebo-controlled Phase 2 study. Br J Cancer. 2021;125(3):366–71. 34006926 10.1038/s41416-021-01356-3 PMC8329046

[68] Xu YJ, et al. Apatinib in patients with extensive-stage small-cell lung cancer after second-line or third-line chemotherapy: a phase II, single-arm, multicentre, prospective study. Br J Cancer. 2019;121(8):640–6. 31523058 10.1038/s41416-019-0583-6 PMC6889407

[69] Cheng Y, et al. Surufatinib plus toripalimab in patients with advanced small cell lung cancer (SCLC) after failure of first-line systemic chemotherapy. Ann Oncol. 2021;32:S1448–9. 10.1016/j.annonc.2021.10.176

[70] Liu YT, et al. A prospective study of apatinib in patients with extensive-stage small cell lung cancer after failure of two or more lines of chemotherapy. Oncologist. 2020;25(5):E833–42. 32250517 10.1634/theoncologist.2019-0391 PMC7216448

[71] Ruan R, et al. Unleashing the potential of combining FGFR inhibitor and immune checkpoint blockade for FGF/FGFR signaling in tumor microenvironment. Mol Cancer. 2023;22(1):60. 36966334 10.1186/s12943-023-01761-7 PMC10039534

[72] Offin M, et al. Tumor mutation burden and efficacy of EGFR-tyrosine kinase inhibitors in patients with EGFR-mutant lung cancers. Clin Cancer Res. 2019;25(3):1063–9. 30045933 10.1158/1078-0432.CCR-18-1102 PMC6347551

[73] Turgeon GA, et al. Radiotherapy and immunotherapy: a synergistic effect in cancer care. Med J Aust. 2019;210(1):47–53. 30636308 10.5694/mja2.12046

[74] Tian YR, et al. Radiation therapy for extensive-stage small-cell lung cancer in the era of immunotherapy. Cancer Lett. 2022;541: 215719. 35597478 10.1016/j.canlet.2022.215719

[75] Wang WW, et al. Abscopal effect of radiation therapy and nivolumab in a patient with combined small-cell lung cancer: a case report. Immunotherapy. 2022;14:909–14. 35787148 10.2217/imt-2021-0050

[76] Welsh JW, et al. Phase 1/2 trial of pembrolizumab and concurrent chemoradiation therapy for limited-stage SCLC. J Thorac Oncol. 2020;15(12):1919–27. 32916308 10.1016/j.jtho.2020.08.022 PMC10600713

[77] Welsh JW, et al. Phase I trial of pembrolizumab and radiation therapy after induction chemotherapy for extensive-stage small cell lung cancer. J Thorac Oncol. 2020;15(2):266–73. 31605794 10.1016/j.jtho.2019.10.001

[78] Spigel DR, et al. Five-year survival outcomes from the PACIFIC trial: durvalumab after chemoradiotherapy in stage III non-small-cell lung cancer. J Clin Oncol. 2022;40:1965. 35108059 10.1200/JCO.21.01308 PMC9015199

[79] Mehra R, et al. Cost-effectiveness of durvalumab after chemoradiotherapy in unresectable stage III NSCLC: a US healthcare perspective. J Natl Compr Canc Netw. 2021;19(2):153–62. 33545688 10.6004/jnccn.2020.7621

[80] Jabbour SK, et al. Pembrolizumab plus concurrent chemoradiation therapy in patients with unresectable, locally advanced, stage III non-small cell lung cancer: the phase 2 KEYNOTE-799 nonrandomized trial. JAMA Oncol. 2021;7(9):1–9. 34086039 10.1001/jamaoncol.2021.2301 PMC8446818

[81] Peters S, et al. Consolidation nivolumab and ipilimumab versus observation in limited-disease small-cell lung cancer after chemo-radiotherapy - results from the randomised phase II ETOP/IFCT 4–12 STIMULI trial. Ann Oncol. 2022;33(1):67–79. 34562610 10.1016/j.annonc.2021.09.011

[82] Yang M, et al. Single-cell transcriptome analysis of radiation pneumonitis mice. Antioxidants. 2022;11(8):1457. 35892659 10.3390/antiox11081457 PMC9331247

[83] Zander DS, Rassaei N. 19 - Drug reactions and other iatrogenic pulmonary diseases. In: Zander DS, Farver CF, editors. Pulmonary pathology (Second Edition). Philadelphia: Elsevier; 2018. p. 396–408.

[84] Roeder F, et al. Correlation of patient-related factors and dose-volume histogram parameters with the onset of radiation pneumonitis in patients with small cell lung cancer. Strahlenther Onkol. 2010;186(3):149–56. 20165822 10.1007/s00066-010-2018-4

[85] Ma J, et al. Bispecific antibodies: from research to clinical application. Front Immunol. 2021;12: 626616. 34025638 10.3389/fimmu.2021.626616 PMC8131538

[86] Keam SJ. Cadonilimab: first approval. Drugs. 2022;82(12):1333–9. 35986837 10.1007/s40265-022-01761-9

[87] Wermke M, et al. Phase I trial of the DLL3/CD3 bispecific T-cell engager BI 764532 in DLL3-positive small-cell lung cancer and neuroendocrine carcinomas. Future Oncol. 2022;18(24):2639–49. 35815644 10.2217/fon-2022-0196

[88] Wermke M, et al. OA01.05 Phase I dose escalation trial of the DLL3/CD3 Igg-Like T cell engager BI 764532 In patients with DLL3+ tumors: focus on SCLC. J Thorac Oncol. 2023;18(11 Supplement):S45–6. 10.1016/j.jtho.2023.09.026

[89] Regzedmaa O, et al. Prevalence of DLL3, CTLA-4 and MSTN expression in patients with small cell lung cancer. Onco Targets Ther. 2019;12:10043–55. 31819500 10.2147/OTT.S216362 PMC6877464

[90] Villaruz LC, et al. Immunotherapy in lung cancer. Transl Lung Cancer Res. 2014;3(1):2–14. 25806276 10.3978/j.issn.2218-6751.2013.10.13 PMC4367610

[91] Grosso F, et al. Pilot study to evaluate serum soluble mesothelin-related peptide (SMRP) as marker for clinical monitoring of pleural mesothelioma (PM): correlation with modified RECIST score. Diagnostics. 2021;11(11):2015. 34829362 10.3390/diagnostics11112015 PMC8623660

[92] Hassan R, Ho M. Mesothelin targeted cancer immunotherapy. Eur J Cancer. 2008;44(1):46–53. 17945478 10.1016/j.ejca.2007.08.028 PMC2265108

[93] Yu L, et al. Mesothelin as a potential therapeutic target in human cholangiocarcinoma. J Cancer. 2010;1:141–9. 20922056 10.7150/jca.1.141 PMC2948219

[94] Ho M, et al. Mesothelin expression in human lung cancer. Clin Cancer Res. 2007;13(5):1571–5. 17332303 10.1158/1078-0432.CCR-06-2161

[95] Grunnet M, Sorensen JB. Carcinoembryonic antigen (CEA) as tumor marker in lung cancer. Lung Cancer. 2012;76(2):138–43. 22153832 10.1016/j.lungcan.2011.11.012

[96] Liu L, et al. The role of human epidermal growth factor receptor 2 as a prognostic factor in lung cancer: a meta-analysis of published data. J Thorac Oncol. 2010;5(12):1922–32. 21155183 10.1097/JTO.0b013e3181f26266

[97] Cui B, et al. Targeting ROR1 inhibits epithelial-mesenchymal transition and metastasis. Cancer Res. 2013;73(12):3649–60. 23771907 10.1158/0008-5472.CAN-12-3832 PMC3832210

[98] Shafer P, Kelly LM, Hoyos V. Cancer therapy with TCR-engineered T cells: current strategies, challenges, and prospects. Front Immunol. 2022;13: 835762. 35309357 10.3389/fimmu.2022.835762 PMC8928448

[99] Chandran SS, Klebanoff CA. T cell receptor-based cancer immunotherapy: emerging efficacy and pathways of resistance. Immunol Rev. 2019;290(1):127–47. 31355495 10.1111/imr.12772 PMC7027847

[100] Lahiri A, et al. Lung cancer immunotherapy: progress, pitfalls, and promises. Mol Cancer. 2023;22(1):40. 36810079 10.1186/s12943-023-01740-y PMC9942077

[101] Chiappori AA, et al. INGN-225: a dendritic cell-based p53 vaccine (Ad.p53-DC) in small cell lung cancer: observed association between immune response and enhanced chemotherapy effect. Expert Opin Biol Ther. 2010;10(6):983–91. 20420527 10.1517/14712598.2010.484801 PMC3146364

[102] Yang X, et al. Synthetic multiepitope neoantigen DNA vaccine for personalized cancer immunotherapy. Nanomedicine. 2021;37: 102443. 34303839 10.1016/j.nano.2021.102443

[103] Muller P, et al. Microtubule-depolymerizing agents used in antibody-drug conjugates induce antitumor immunity by stimulation of dendritic cells. Cancer Immunol Res. 2014;2(8):741–55. 24916470 10.1158/2326-6066.CIR-13-0198

[104] Zamarin D, et al. Localized oncolytic virotherapy overcomes systemic tumor resistance to immune checkpoint blockade immunotherapy. Sci Transl Med. 2014. 10.1126/scitranslmed.3008095. 24598590 10.1126/scitranslmed.3008095 PMC4106918

[105] Bommareddy PK, Shettigar M, Kaufman HL. Integrating oncolytic viruses in combination cancer immunotherapy. Nat Rev Immunol. 2018;18(8):498–513. 29743717 10.1038/s41577-018-0014-6

[106] Kellish P, et al. Oncolytic virotherapy for small-cell lung cancer induces immune infiltration and prolongs survival. J Clin Investig. 2019;129(6):2279–92. 31033480 10.1172/JCI121323 PMC6546459

[107] Fukuhara H, Ino Y, Todo T. Oncolytic virus therapy: a new era of cancer treatment at dawn. Cancer Sci. 2016;107(10):1373–9. 27486853 10.1111/cas.13027 PMC5084676

[108] Owen DH, et al. DLL3: an emerging target in small cell lung cancer. J Hematol Oncol. 2019;12:1–8. 31215500 10.1186/s13045-019-0745-2 PMC6582566

[109] Rudin CM, et al. Emerging therapies targeting the delta-like ligand 3 (DLL3) in small cell lung cancer. J Hematol Oncol. 2023;16(1):66. 37355629 10.1186/s13045-023-01464-y PMC10290806

[110] Saunders LR, et al. A DLL3-targeted antibody-drug conjugate eradicates high-grade pulmonary neuroendocrine tumor-initiating cells in vivo. Sci Transl Med. 2015;7(302): 302136. 10.1126/scitranslmed.aac9459 PMC4934375 26311731

[111] Rudin CM, et al. Rovalpituzumab tesirine, a DLL3-targeted antibody-drug conjugate, in recurrent small-cell lung cancer: a first-in-human, first-in-class, open-label, phase 1 study. Lancet Oncol. 2017;18(1):42–51. 27932068 10.1016/S1470-2045(16)30565-4 PMC5481162

[112] Paz-Ares L, et al. Tarlatamab, a first-in-class DLL3-targeted bispecific T-cell engager, in recurrent small-cell lung cancer: an open-label, phase I study. J Clin Oncol. 2023;41(16):2893–903. 36689692 10.1200/JCO.22.02823 PMC10414718

[113] Valenzuela LB, et al. Anti-tumor activity of HPN328, a DLL3-targeting tri-specific, half-life extended T cell engager, is enhanced by combining with an anti-PD-L1 antibody in an immunocompetent mouse model. Cancer Res. 2023;83(7):5070. 10.1158/1538-7445.AM2023-5070

[114] Chauvin JM, Zarour HM. TIGIT in cancer immunotherapy. J Immunother Cancer. 2020;8(2): e000957. 32900861 10.1136/jitc-2020-000957 PMC7477968

[115] Zhang Q, et al. Blockade of the checkpoint receptor TIGIT prevents NK cell exhaustion and elicits potent anti-tumor immunity. Nat Immunol. 2018;19(7):723. 29915296 10.1038/s41590-018-0132-0

[116] Lucca LE, et al. TIGIT signaling restores suppressor function of Th1 Tregs. Jci Insight. 2019. 10.1172/jci.insight.124427. 30728325 10.1172/jci.insight.124427 PMC6413794

[117] Anderson AC, Joller N, Kuchroo VK. Lag-3, Tim-3, and TIGIT: co-inhibitory receptors with specialized functions in immune regulation. Immunity. 2016;44(5):989–1004. 27192565 10.1016/j.immuni.2016.05.001 PMC4942846

[118] Mettu NB, et al. A phase 1a/b open-label, dose-escalation study of etigilimab alone or in combination with nivolumab in patients with locally advanced or metastatic solid tumors. Clin Cancer Res. 2022;28(5):882–92. 34844977 10.1158/1078-0432.CCR-21-2780

[119] Arcaro A. Targeted therapies for small cell lung cancer: where do we stand? Crit Rev Oncol Hematol. 2015;95(2):154–64. 25800975 10.1016/j.critrevonc.2015.03.001

[120] Wei J, et al. Clinicopathological features and prognostic implications of ASCL1 expression in surgically resected small cell lung cancer. Thorac Cancer. 2021;12(1):40–7. 33191657 10.1111/1759-7714.13705 PMC7779202

[121] Huang W, et al. Combination therapy: future directions of immunotherapy in small cell lung cancer. Transl Oncol. 2021;14(1): 100889. 33065386 10.1016/j.tranon.2020.100889 PMC7567053

[122] Wang S, et al. Efficacy and safety of first-line immune checkpoint inhibitors combined with chemotherapy for extensive-stage small cell lung cancer: a network meta-analysis. Lung Cancer. 2023;178:47–56. 36774774 10.1016/j.lungcan.2023.02.003

[123] Bilani N, et al. Effect of immunotherapy on overall survival in limited-stage small cell lung carcinoma: a national cancer database analysis. Ther Adv Med Oncol. 2021;13:1758835920982806. 33747146 10.1177/1758835920982806 PMC7905481

[124] Li T, Qiao T. Unraveling tumor microenvironment of small-cell lung cancer: implications for immunotherapy. Semin Cancer Biol. 2022;86(Pt 2):117–25. 36183998 10.1016/j.semcancer.2022.09.005

[125] Niu X, et al. Ferroptosis, necroptosis, and pyroptosis in the tumor microenvironment: perspectives for immunotherapy of SCLC. Semin Cancer Biol. 2022;86(Pt 3):273–85. 35288298 10.1016/j.semcancer.2022.03.009

[126] Pirlog R, et al. Cellular and molecular profiling of tumor microenvironment and early-stage lung cancer. Int J Mol Sci. 2022;23(10):5346. 35628157 10.3390/ijms23105346 PMC9140615

[127] Lei X, et al. Immune cells within the tumor microenvironment: biological functions and roles in cancer immunotherapy. Cancer Lett. 2020;470:126–33. 31730903 10.1016/j.canlet.2019.11.009

[128] Pakkala S, et al. Durvalumab and tremelimumab with or without stereotactic body radiation therapy in relapsed small cell lung cancer: a randomized phase II study. J Immunother Cancer. 2020;8(2): e001302. 33428583 10.1136/jitc-2020-001302 PMC7754662

[129] Zheng Z, et al. Advances in new targets for immunotherapy of small cell lung cancer. Thorac Cancer. 2024;15(1):3–14. 38093497 10.1111/1759-7714.15178 PMC10761621

[130] Solta A, et al. Small cells - big issues: biological implications and preclinical advancements in small cell lung cancer. Mol Cancer. 2024;23(1):41. 38395864 10.1186/s12943-024-01953-9 PMC10893629

[131] Mamdani H, et al. Immunotherapy in lung cancer: current landscape and future directions. Front Immunol. 2022;13: 823618. 35222404 10.3389/fimmu.2022.823618 PMC8864096

[132] Yuan M, et al. Signal pathways and precision therapy of small-cell lung cancer. Signal Transduct Target Ther. 2022;7(1):187. 35705538 10.1038/s41392-022-01013-y PMC9200817

[133] Demedts IK, Vermaelen KY, van Meerbeeck JP. Treatment of extensive-stage small cell lung carcinoma: current status and future prospects. Eur Respir J. 2010;35(1):202–15. 20044461 10.1183/09031936.00105009

[134] Zhang S, Cheng Y. Immunotherapy for extensive-stage small-cell lung cancer: current landscape and future perspectives. Front Oncol. 2023. 10.3389/fonc.2023.1142081. 37188176 10.3389/fonc.2023.1142081 PMC10175664

